# The current state of the screening and enrollment pipeline for clinical research engagement in the era of disease modifying therapies for Alzheimer’s disease

**DOI:** 10.1002/alz70859_106302

**Published:** 2025-12-26

**Authors:** Elif PINAR Coskun, Melanie G Elam, Laura Clewett, Lauren Bojarski, Alexandra Curtis, Molly Harper, Rosmy George, Andrea Shaffer, Muna Amry, Shreeya Shrestha, Gregory A Jicha

**Affiliations:** ^1^ University of Kentucky, SBCoA, Lexington, KY USA; ^2^ University of kentucky, Department of Neurology, Lexington, KY USA; ^3^ University of Kentucky, Sanders Brown Center on Aging, Lexington, KY USA; ^4^ University of Kentucky College of Medicine Department of Neurology, Lexington, KY USA; ^5^ University of Kentucky College of Medicine Sanders‐Brown Center on Aging, Lexington, KY USA; ^6^ Sanders‐Brown Center on Aging, Lexington, KY USA; ^7^ University of Kentucky, Lexington, KY USA; ^8^ University of kentuckyUniversity of Kentucky, Sanders Brown Center on Aging, Lexington, KY USA; ^9^ University of Kentucky Sanders‐Brown Center on Aging, Lexington, KY USA; ^10^ University of Kentucky College of Medicine, Lexington, KY USA

## Abstract

**Background:**

Recruitment and engagement are major challenges for clinical trials in the area of Alzheimer’s disease and related dementias (ADRD). This problem may be exacerbated by the recent FDA approval of anti‐amyloid therapies and their introduction into standard clinical practice. A better understanding of the barriers to research engagement may increase research productivity resulting in shorter trials that can move the needle forward at a faster rate. This study sought to explore the pipeline from prescreening participants for clinical trials of mild cognitive impairment (MCI) and early Alzheimer’s disease (AD) in order to provide insights into the major current barriers for research engagement in this era of disease modifying therapies.

**Method:**

The present data analyzes the recruitment and engagement pipeline for two current clinical trials of experimental medications for the treatment of MCI and early AD. Descriptions of reasons for screen failure along the pipeline are presented based on summary categorization in order to better understand the barriers to clinical trial recruitment at present.

**Result:**

Over the last year, 490 potential research participants were prescreened for clinical trial engagement, with 45 moving on to consent (<10%), and only 8 of these eventually being enrolled (<20%), demonstrating an overall success rate for clinical trial engagement at only 1.6% in the current study. Approximately 23% of participants were to clinical trial engagement, and another 20% of potential participants failed to engage in pursuit of alternate treatments, including nearly 10% due to pursuit of recently approved anti‐amyloid therapy treatments. Thirty‐eight percent of potential participants screen‐failed for medical conditions and exclusionary medications, of which nearly one‐half were not related to safety and or primary outcome measures. Less than 5% of potential participants could not participate due to trial logistics (travel, need for a study partner, duration and or frequency of visits).

**Conclusion:**

This data suggests that increasing the pipeline of potential research participants for clinical trials should focus on continued efforts to destigmatize clinical trial engagement, adapt inclusion/exclusion criteria to allow concomitant disease‐modifying therapies, and eliminate unnecessary exclusion criteria if it does not affect safety and or primary outcome measures.

